# Global research trends in tumor-associated macrophage studies: a bibliometric analysis

**DOI:** 10.1007/s12672-025-02473-8

**Published:** 2025-05-09

**Authors:** Aina Luan, Yipeng Zhang, Lu Yang, Guojing Zhao, Xin Yang

**Affiliations:** 1 Department of Geriatric Psychiatry, Chongqing Eleventh People’s Hospital, Chongqing, 400038 People’s Republic of China; 2 Chongqing Key Laboratory of Translational Research for Cancer Metastasis and Individualized Treatment, Chongqing University Cancer Hospital, No. 181 of Hanyu Road, Shapingba District, Chongqing, 400030 People’s Republic of China

**Keywords:** Bibiliometric study, Citespace, Developmental trends, R software, Hot spots, TAMs

## Abstract

**Objectives:**

Macrophages play a critical role in various diseases, including cancer, where their involvement is characterized by a dual nature. There is a growing focus on tumor-associated macrophages (TAMs) in cancer research due to their complex interactions with tumor biology. With the expanding body of research in this area, a retrospective analysis of published articles is warranted to gain insights into evolving trends. This bibliometric study aims to assist researchers in identifying key areas of interest and emerging directions within the field of TAM research.

**Methods:**

A bibliometric analysis was performed using the Bibliometrix Package in R Software and CiteSpace software.

**Results:**

The volume of research on TAMs continues to increase, with this study identifying major contributors to the field. The focus of research has shifted from traditional methods, such as flow cytometry and histological techniques, toward single-cell omics approaches, which offer unbiased insights into TAM heterogeneity. Current areas of interest include biomarkers, immune therapies, TAM states, tumor microenvironments, macrophage-targeted agents, and the response of TAMs to therapeutic interventions. These topics are anticipated to remain prominent in the near future.

**Conclusion:**

The study provides an overview of annual publication trends, influential papers, key journals, frequently used keywords, leading authors, and contributing institutions. It also highlights the interdisciplinary evolution of TAM-related research and the connections between these areas of study.

**Supplementary Information:**

The online version contains supplementary material available at 10.1007/s12672-025-02473-8.

## Introduction

Macrophages are critical immune cells involved in maintaining homeostasis, regulating inflammation, promoting wound healing, facilitating tissue repair, and contributing to tumor progression [1–5]. They reside in all tissues, where they recognize potential pathogens and help prevent disease by phagocytosing and digesting cellular debris, viruses, bacteria, senescent cells, cancer cells, and other foreign matter [6, 7]. There is a double-edged relationship between macrophages and cancer [8]. In some cases, the presence of macrophages can indicate a good prognosis; for instance, high frequencies of HLA-DR^+^ macrophages within tumors have been correlated with positive prognoses [9–11]. Early studies demonstrated that bacterial products and cytokines could activate and augment macrophages, enhancing their ability to eliminate cancer cells and inhibit tumor growth [12, 13].

However, macrophages also contribute to the significant infiltration of leukocytes in most tumor tissues. When exposed to the tumor microenvironment (TME), macrophages are frequently induced to produce immunosuppressive tumor-associated macrophages (TAMs) [14]. TAMs play a central role in cancer progression and metastasis, and their abundance is often linked to poor clinical outcomes [15].

TAMs are characterized by a continuum of phenotypes, ranging from immunostimulatory M1-like to immunosuppressive M2-like states [16, 17]. In the early stages of cancer, macrophages with M1-like characteristics predominate, exerting anti-tumor effects by directly killing cancer cells and activating anti-tumor T cells [8, 18–21]. As the tumor progresses, cues from the TME can drive macrophages toward an M2-like phenotype [22–24]. The majority of macrophages within established tumors are M2-like, inducing immunosuppression, promoting angiogenesis, and inhibiting tumor cell proliferation [8, 18].

Overall, macrophages significantly influence tumor immunity. As research advances, TAMs have garnered increasing attention. Therefore, a retrospective analysis of the published articles in this field is warranted. A bibliometric analysis can provide an overview of research developments, guiding researchers toward key areas of focus and emerging research directions.

As a subfield of informatics, bibliometrics analyzes and interprets patterns in scientific literature to gain insights into emerging trends and knowledge structures within a specific subject area. This method enables an objective evaluation of the current state of a discipline and facilitates the tracking of its progress over time. By utilizing bibliometric analysis software, researchers can develop knowledge maps that outline key research dimensions [25]. Typically, scientific publications are used as input to generate interactive visual representations of complex structures for statistical analysis and interactive exploration. Through these visualizations, the primary research topics within a field can be identified by mapping and analyzing a large body of articles [26, 27].

This study aims to perform a bibliometric analysis of the field of TAMs using bibliometric software and predefined indicators. In addition, this study will visualize, analyze, and plot the tumor-related macrophage field, providing a detailed analysis of its current state, trends, and potential areas for future development.

## Sources and methods

**Fig. 1:**
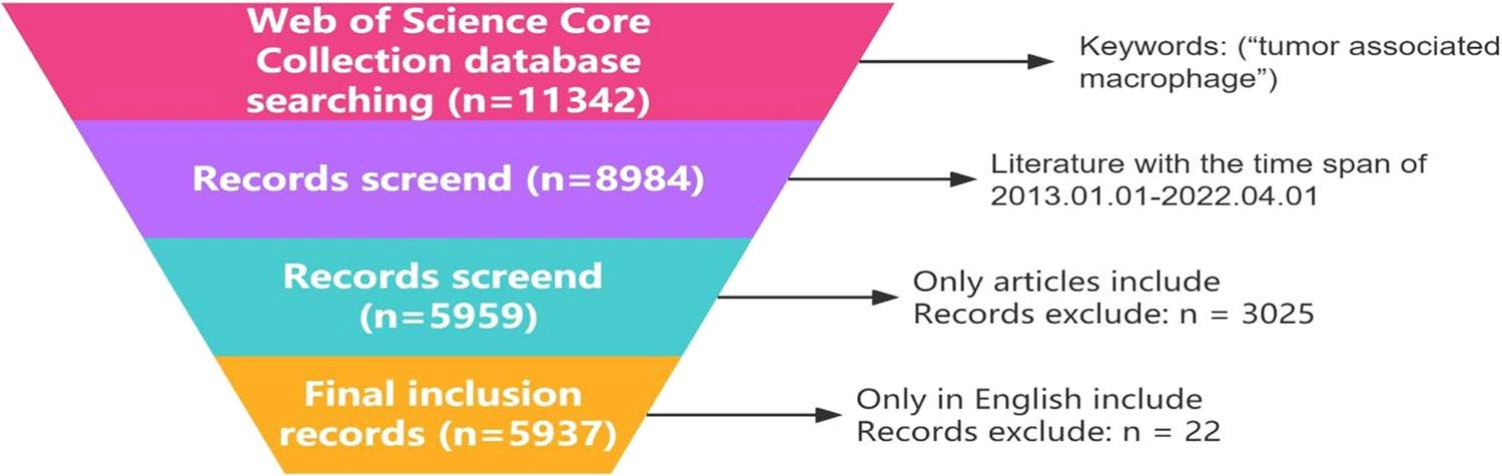
Data selection process

The Web of Science is a comprehensive resource providing access to literature across the hard sciences, social sciences, art, and humanities, and it also serves as a global citation database for leading academic publishers. For this study, the following index keywords were used to search for relevant articles in the Web of Science Core Collection database: “tumor associated macrophages”, SCI-EXPANDED, SSCI, A&HCI, ESCI, CCR-EXPANDED, IC and literature resource. The search covered the time period from January 1st, 2013 to June 1st, 2022 and included only English-language articles. Totally, 5937 documents were retrieved based on this search method and subjected to bibliometric analysis. Figure 1 illustrates the four-step filtering process applied to these documents.

Several approaches were employed to present the bibliographic data. The number of productions and citations (including total citations and average citations per paper) were selected as the primary bibliometric indicators, as they reflect research productivity and influence, respectively [28, 29]. Additionally, other standard measures were used, including international collaborations, contributions from top journals, authors, countries, institutions, and the historical direct citation network [30–32]. A dual-map analysis was also conducted to illustrate the relationships between different disciplines. For analysis purposes, data were downloaded in various formats and processed using R Studio and CiteSpace (versions 4.0.3.1 and 5.7.R5W, respectively) [33]. The R package Bibliometrix was utilized to extract basic information, such as annual trends in scientific productivity, keyword frequency and density visualizations, international collaborations, institutional contributions, and author achievements over time, along with historical direct citation networks [33]. CiteSpace was further used to create dual-map overlays depicting the correlation between disciplines in TAM research and to identify prominent research areas between 2013 and 2022.

## Results

### Global publication trends

**Fig. 2:**
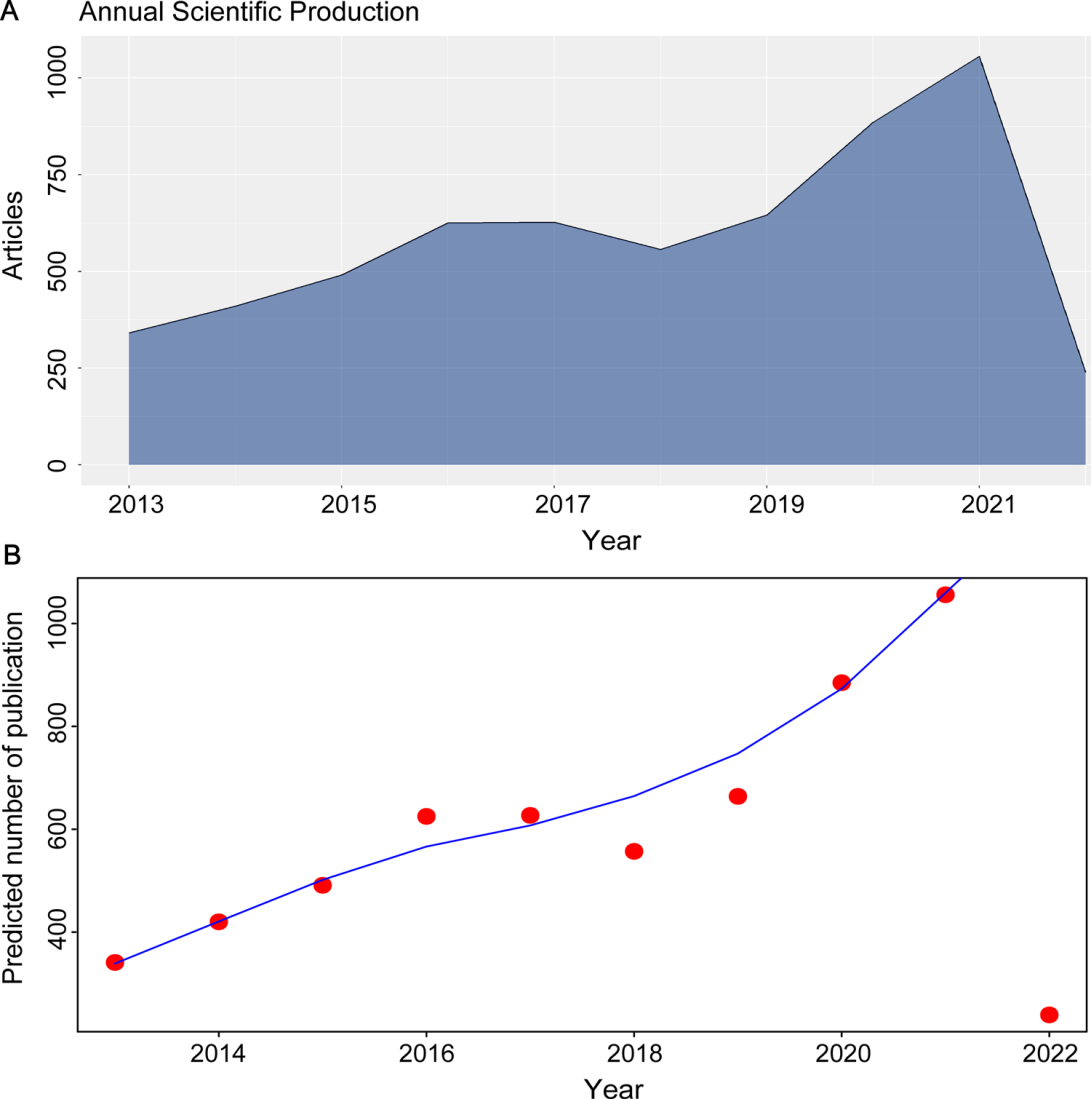
Trends in TAM publications from 2013 to 2022. **a** Annual publication volume, **b** Growth trend curves

A comprehensive list of articles related to TAMs was compiled for the period 2013–2022. As illustrated in Fig. 2a, the number of publications in this field has shown a consistent upward trend each year, with the exception of a slight decline in 2018. Over the past 3 years (starting from 2019), the annual publication volume exceeded 600 articles, reaching 1056 in 2021. From 2019 to 2021, an exponential growth trend in publication output was observed. By the end of March 2022, 239 articles had already been published, with more anticipated throughout the year. In 2022, the total number of publications was expected to surpass 1200. TAMs is a field that has gained much attention from scholars, as evidenced by an increase in articles published annually. A logistic regression model further supports the observation of rapid growth in this field (see Fig. 2b).

### Distribution and cooperation among nations and organizations

**Fig. 3:**
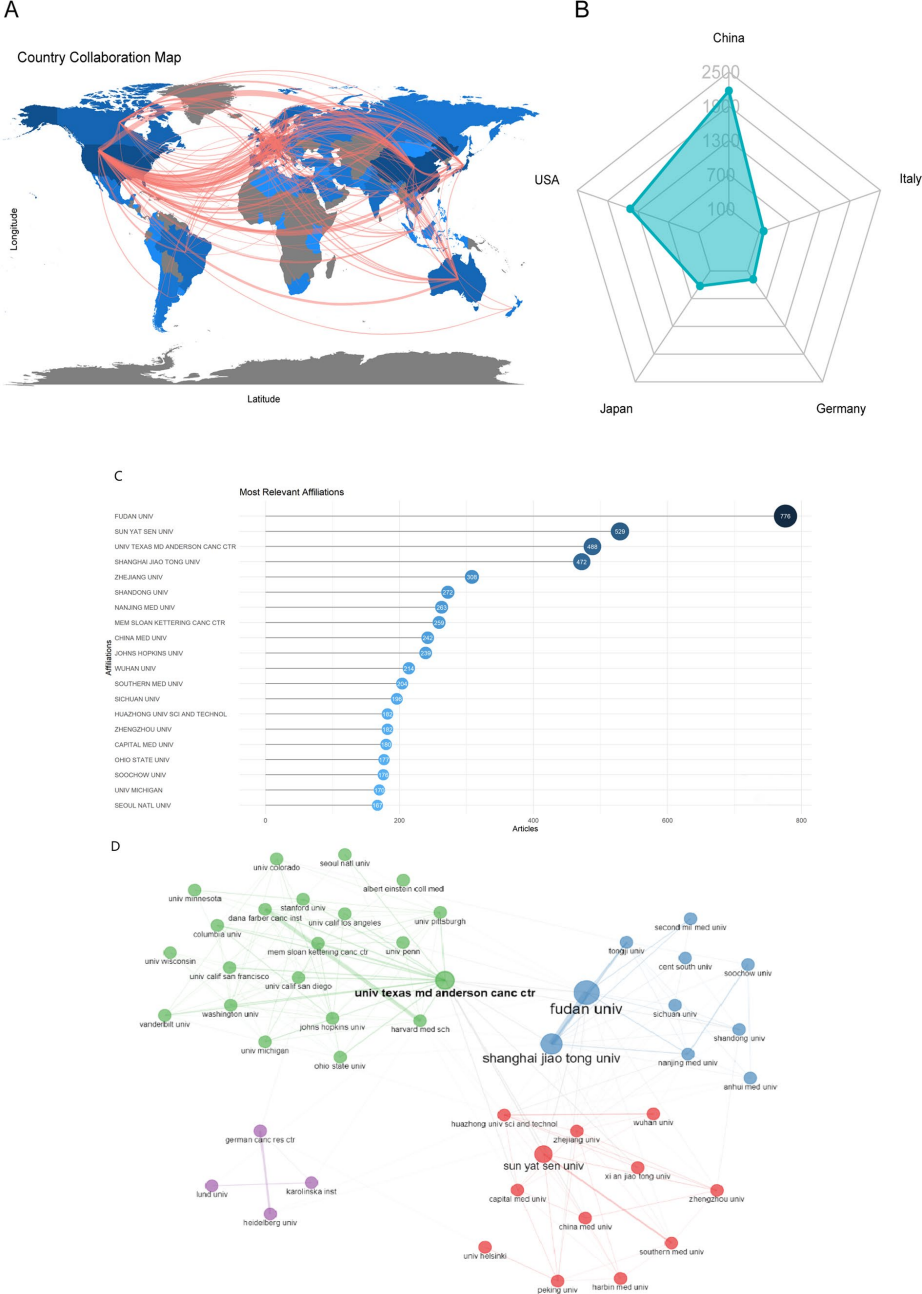
Distribution of country and institutional productivity. **a** Global collaboration map; **b** Country publication radar map; **c** Institutional publication volume; **d** Institutional collaboration graph

A blue-coded world map generated using R software (see Fig. 3a) illustrates the contributions of various countries to TAMs research. China (15), England (23), Germany (158), France (58), and the United States (587 times) had the strongest overall link strength. Publications in this area originated from 84 countries and regions, with China contributing the largest share of articles (2169 articles, representing 36.56% of the total). The United States followed with 1452 articles (24.48%), Japan with 425 (7.16%), Germany with 280 (4.72%), and Italy with 188 (3.17%) (see Table 1, Fig. 3b). In terms of citations, the most cited studies were from the United States (49,585 citations), followed by China (34,741), Japan (8342), Germany (7800), and Italy (4710) (see Table 1).

**Table 1:** Top 20 productive countries and their total and average citations concerning TAMs

Country	Production (articles)	Total citations	Average citations
China	2169	34,741	16.02
USA	1452	49,585	34.15
Japan	425	8342	19.63
Germany	280	7800	27.86
Italy	188	4710	25.05
Korea	169	2879	17.04
United Kingdom	118	4084	34.61
France	111	2744	24.72
Netherlands	94	2349	24.99
Sweden	87	2366	27.2
Canada	71	1519	21.39
Australia	65	1145	33.68
Spain	62	1627	26.24
Switzerland	61	2016	33.05
Israel	47	1519	32.32
Belgium	43	1802	41.91
Finland	39	1147	29.41
Poland	37	439	11.86
India	35	507	14.49
Austria	34	1145	33.68

TAMs, tumor-associated macrophages

A total of 5830 institutions contributed to TAMs research. As shown in Fig. 3c, most publications came from Fudan University (776 records, 13.07%), Sun Yat-sen University in Guangzhou (529, 8.91%), the M.D. Anderson Cancer Center at the University of Texas (488, 8.21%), Shanghai Jiao Tong University (472, 7.95%), and Zhejiang University (308, 5.19%).

Figure 3d illustrates the collaborative relationships among institutions. A total of 47 institutions were identified, and four distinct clusters were categorized, each represented by a different color. The green cluster, comprising 21 institutions is the largest, and primarily includes institutions based in the United States, such as the University of Texas M.D. Anderson Cancer Center and Memorial Sloan-Kettering Cancer Center. The second largest cluster, depicted in red, consists of 11 institutions, predominantly from China, with Sun Yat-sen University and Zhejiang University leading the group. The size of each node represents the degree of collaboration centralization, while the thickness of the connecting edges indicates the strength of the collaborative relationships.

### Top authors’ contributions over time

**Fig. 4:**
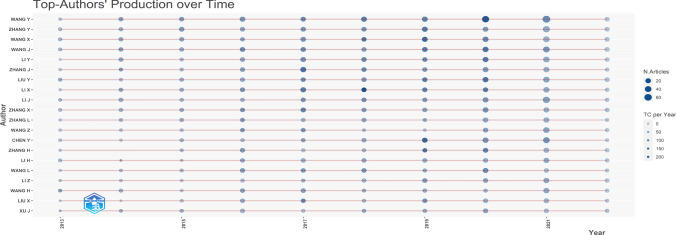
Production of the top 20 authors over time

The number of publications and citation frequency are critical indicators for assessing an author’s influence in a given field. A higher volume of published work and citation rates typically signify greater authority within the domain. Figure 4 presents the top 20 authors in the domain of tumor-related macrophage research over time. Size of nodes corresponds to the number of publications, while the depth of the node’s color corresponds to the number of citations per year. Wang Y. has the highest number of publications and citations from 2013 to 2022, followed by Zhang Y.

### Journal sources

This analysis evaluated the local impact of source journals based on their H-index, which reflects both publication volume and citation impact (see Table 2). “Cancer Research” is one of the most influential journals, with an H index of 66, a total of 15,767 citations, and 280 publications, primarily focusing on tumor-related research. This is followed by “Clinical Cancer Research” (H-Index 56, 8815 citations, 181 publications) and “Oncotarget” (H-Index 52, 11,745 citations, 413 publications). These findings indicate that studies on TAMs are primarily published in US—based oncology journals. These journals contain information about macrophages, making them relevant for researchers interested in tumor biology. As a result, the three local sources most likely to be cited are “Cancer Research,” “Clinical Cancer Research,” and “Oncotarget.” The resources provided scholars with reference material for further research on TAMs and contributed to the intellectual foundation of this field.

**Table 2:** Top 20 most productive and impact journals concerning TAMs

Journal	H_index	Total citation	Publication (articles)
Cancer Research	66	15,767	280
Clinical Cancer Research	56	8815	181
Oncotarget	52	11,745	413
Oncogene	43	5837	136
Oncoimmunology	41	6701	265
Cancer Immunology Research	40	5383	127
International Journal of Cancer	37	4133	119
British Journal of Cancer	34	3058	72
Cancer Letters	34	3428	138
Cancer Cell	32	7383	49
BMC Cancer	27	2745	128
Journal for immunotherapy of Cancer	27	2746	156
Cancer Science	25	2302	91
Cancer Immunology Immunotherapy	24	2092	134
Journal of Experimental and Clinical Cancer Research	24	1801	52
Oncology Reports	23	1461	95
Tumor Biology	23	1427	69
Breast Cancer Research	22	1308	36
Cancer Discovery	22	2840	30
Molecular Cancer Therapeutics	22	1366	58

TAMs, tumor-associated macrophages

### Analysis of discipline association using dual-maps

A bipartite overlay is a visual analysis method where two maps are superimposed. The first is referred to as the overlay map, and the second is the base map. By comparing map overlays and their results, valuable insights into the data sources can be uncovered [26, 34–36]. The base map in this context includes over 10,000 cited journals from the Web of Science (WoS) database, while the overlay map is derived from the data of the cited literature. By comparing these maps, insights can be gained into the relationships between research topics and their data sources [37–39]. Thus, dual-map overlays offer a broader perspective on the evolution of content at the discipline level.

**Fig. 5:**
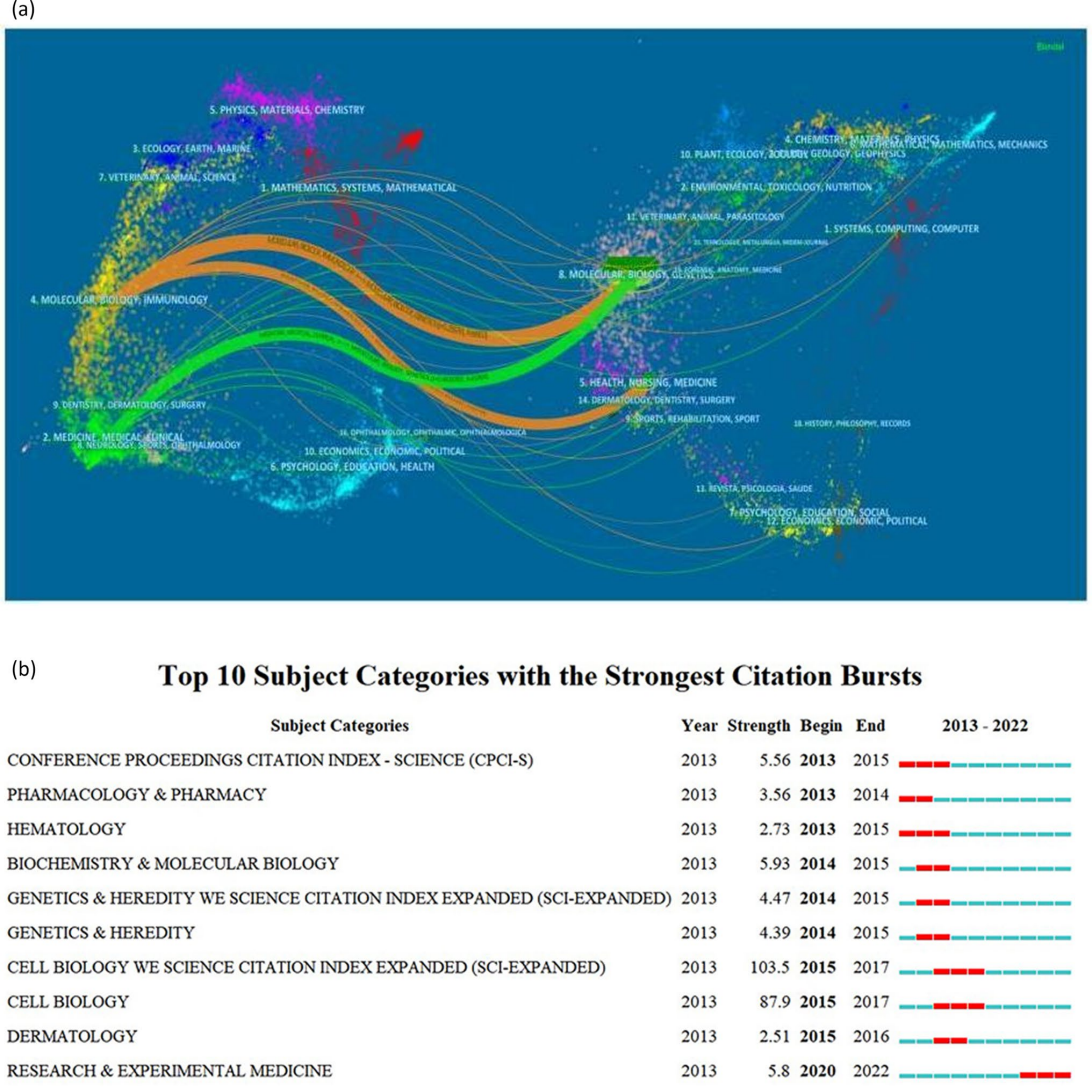
**a** A dual-map overlay showing the correlation between disciplines in TAM research; **b** Burst disciplines in TAM research from 2013 to 2022

The present study utilizes the Journal Citation Reports (JCR) data to generate CiteSpace overlay maps, allowing for the integration of TAMs research data onto the base map. Figure 5a visualizes the associations between disciplines in tumor-related macrophage research. On the left-hand side of the figure, the current status of research in disciplines related to TAM is depicted, while the right side represents the foundational research disciplines based on cited literature. Present research and research base are linked by the wave-shaped curve [40]. Two major areas of focus emerge: “Molecular, Biology, and Immunology,” with “Medicine, Medical, and Clinical” on the left, and “Molecular, Biology, Genetics,” and “Health, Nursing, and Medicine” on the right. The majority of published papers appear on the left side, influenced by the foundational work on the right. Citations follow three main trails that are depicted as green, and orange curves highlighted with journal tags.

### Citation bursts of disciplines

The table of discipline types from 2013 to 2022 as shown in Fig. 5b, indicates that the field of TAM experiences bursts of disciplines on an annual basis. However, these emerging disciplines tend to be short-lived. The most sustained bursts, lasting approximately 3 years, were observed in the fields of the Conference Proceedings Citation Index–Science, Hematology, Cell Biology, and Research & Experimental Medicine. This prolonged interest is likely due to significant developments in the stages of TAM research, attracting increased attention from tumor immunologists. The integration of research hotspots in these disciplines may further accelerate the growth of this field.

### Historical evolution of TAM research

**Fig. 6:**
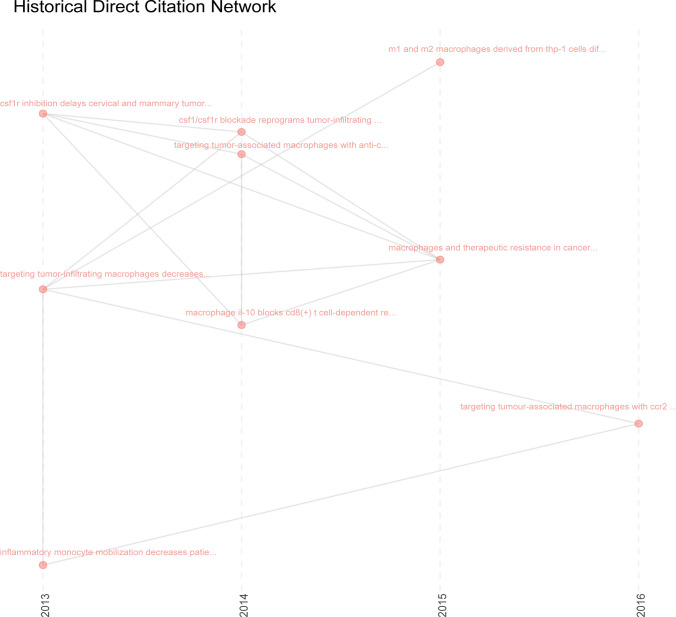
Direct citation network showing historical trends in TAM research, with links representing citation relationships

The historical direct citation network for TAM research was analyzed to uncover systemic shifts in the focus of this research over time (see Fig. 6). Macrophage studies across various cancer types have progressed at different stages. Since work on the project began in 2013, attention has been directed toward the anti-tumor effects of macrophages under different conditions. Thereafter, tumor-related macrophages as a strategy for cancer therapy was given more attention in 2014. In 2015, research largely focused on M1/M2 phenotypic differentiation of macrophages in cancer therapy. As the process advanced, by 2016, scientists began to express concern about the tumor-promoting role of TAMs in progression of cancers. In 2019, further discoveries highlighted the roles of TAM in cancer-specific reprogramming, the identification of biomarkers, and therapeutic targeting.

### Keyword analysis

**Fig. 7:**
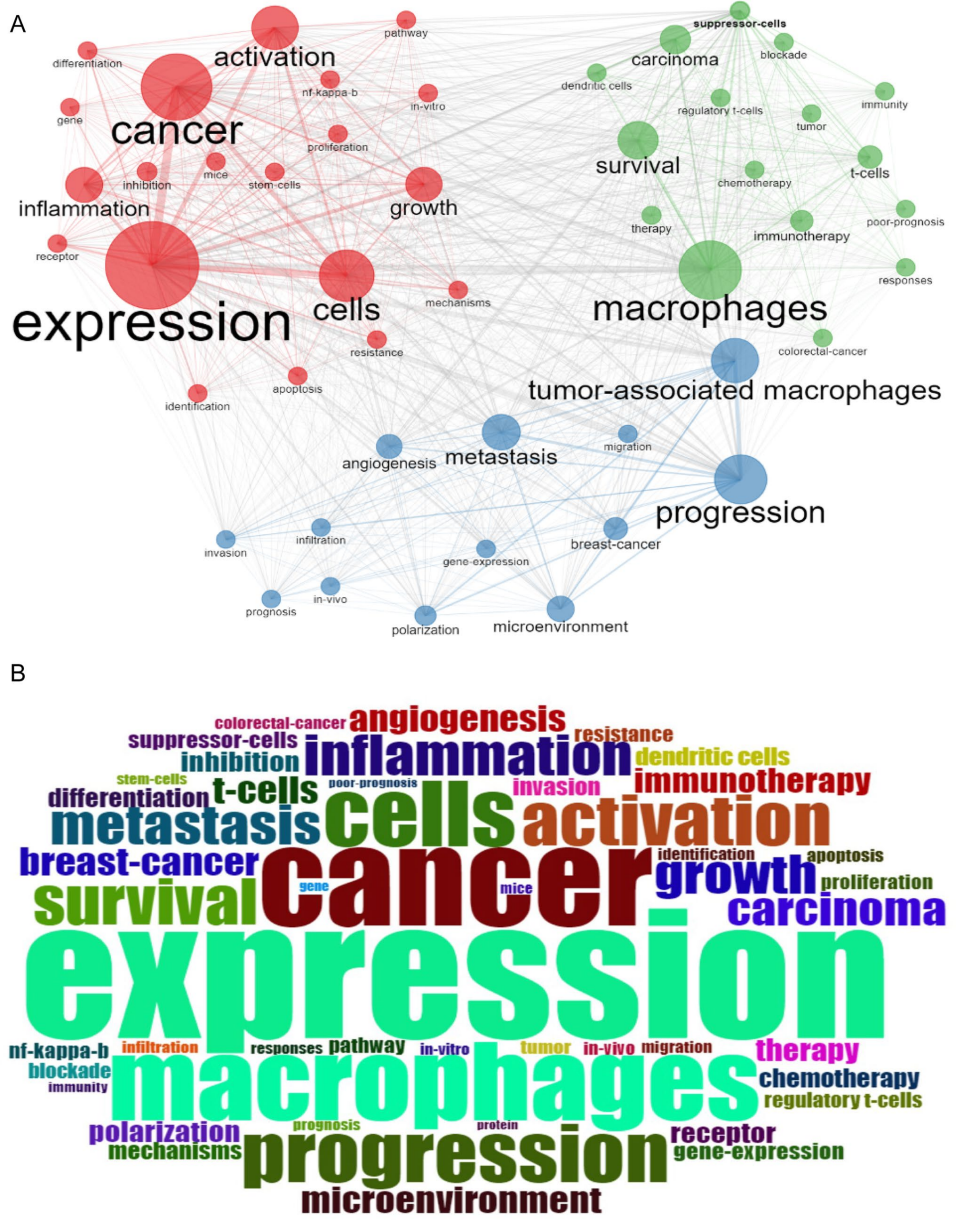
Keyword analysis. **a** Density visualization of keywords; **b** Keyword frequency trends

The analysis of research hotspots in TAM was conducted through a density visualization based on keyword frequency from 2013 to March 2022. Higher-frequency keywords were visualized as denser nodes, indicating areas of significant research focus. Figure 7a presents three primary keyword clusters.

1. Cluster 1 (red) dealt primarily with expression and mechanism, including expression, inflammation, activation, differentiation, pathway, as well as inhibition.
2. Cluster 2 (blue) is comprised of the disease course, including TAMs, progression, metastasis, and growth.
3. Cluster 3 (green) contained immune, therapy, and survival with keywords like macrophage, regulatory T-cell, dendritic cell, blockade, immunity, immunotherapy, chemotherapy, prognosis, and poor prognosis.

Tracking the frequency of these keywords as shown in Fig. 7b, highlights major research priorities within TAM. Commonly, the topics studied include “expression,” “macrophages,” “cancer,” “cell,” “progression,” “activation,” “inflammation,” and “microenvironment.” These areas reflect the key themes and efforts of scholars within the field.

### Keyword burst

**Fig. 8:**
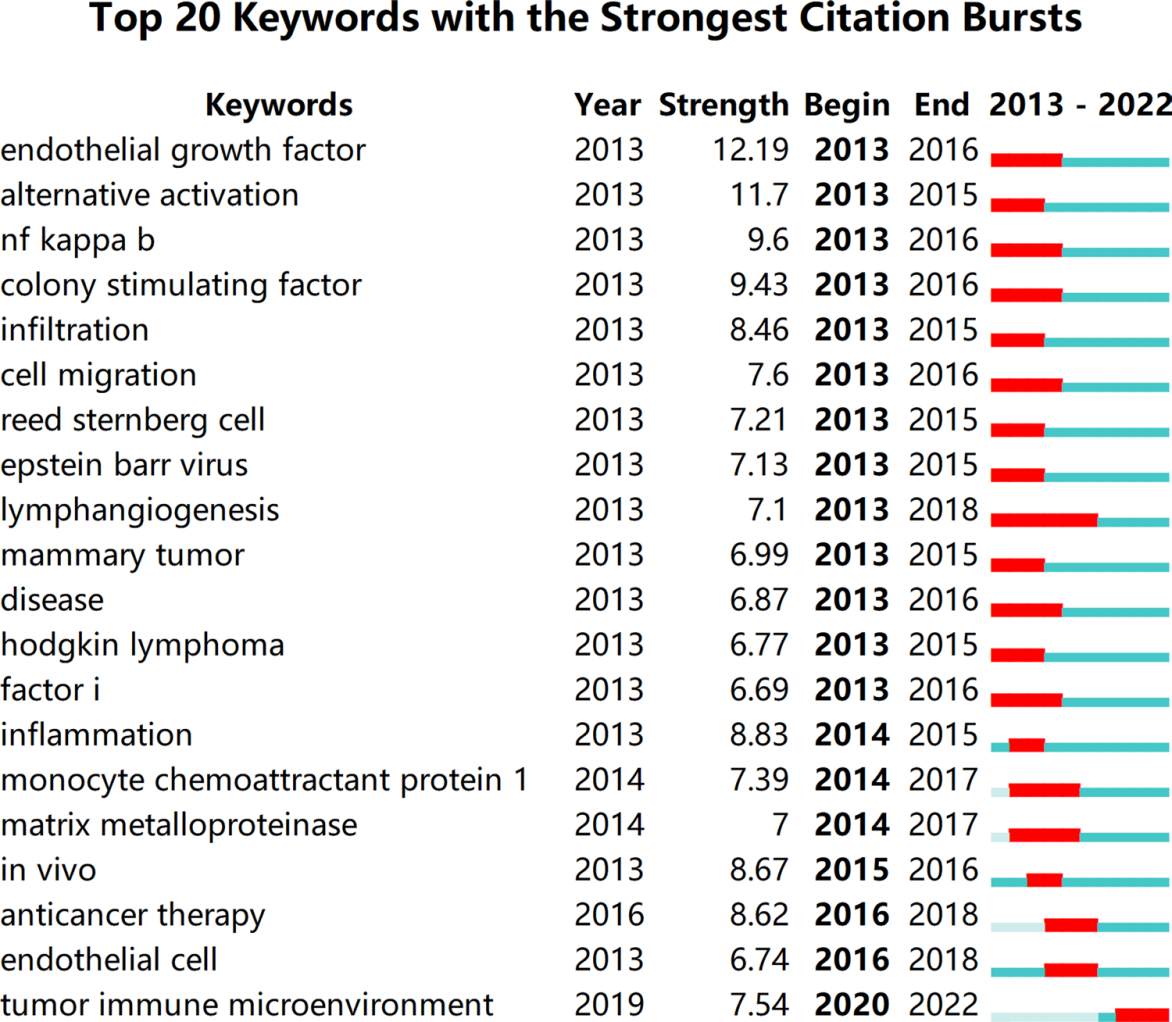
Top 20 Keywords with the strongest citation bursts

Between 2013 and 2022, keyword burst analysis identified key research trends in the study of TAMs. Figure 8 presents the top 20 keywords exhibiting the highest burst intensities. “Vascular endothelial growth factor (VEGF)” demonstrated significant activity from 2013 to 2016, with a burst intensity of 12.19. Similarly, “alternative activation” was prominent from 2013 to 2015, with an intensity of 11.7, while “NF-κB” showed sustained activity from 2013 to 2016, reaching an intensity of 9.6. The term “tumor immune microenvironment” remained highly active from 2020 to 2022, indicating its emergence as a central focus in the field.

A fundamental association exists between TAMs and the tumor immune microenvironment (TIME), where TAMs constitute a critical component. The polarization states of TAMs, categorized as M1-like and M2-like macrophages, influence their functional roles in tumor progression. M1-like macrophages generally exert anti-tumor effects, whereas M2-like macrophages contribute to tumor growth, metastasis, and immune evasion. Through the secretion of various cytokines and chemokines, TAMs regulate the activity of other immune cells, thereby shaping the TME. This understanding has positioned TAM-targeted therapeutic strategies as a promising direction in cancer research. Insights into TAM-TIME interactions may contribute to the development of novel cancer therapies and improved treatment outcomes.

### Author-based keyword clusters

The interconnections among these keywords give rise to distinct clusters based on their thematic associations. The identification of these clusters offers a structured perspective on research trends in the field of TAMs. Additional file 1: Fig. 1 presents eight distinct clusters: #0 immunotherapy, #1 pan-cancer, #2 adjuvant chemotherapy, #4 triple-negative breast cancer, #5 esophageal squamous cell carcinoma, #6 malignant pleural effusion, #7 anti-PD-1, and #9 sialic acid. These clusters highlight key research areas within TAM studies and the contributions of leading researchers in the field. Within the #0 immunotherapy cluster, Wei Zhang and Wei Wang et al. have been identified as prominent contributors to scientific collaboration.

## Discussion

This study conducted a comprehensive analysis of the literature on TAMs from its inception, using CiteSpace metrological analysis software and the Bibliometrix package in R to quantitatively and visually assess research progress in this area. The quantitative analysis encompassed fundamental metrics, including annual publication numbers, authors, and country of origin, journals, and institutions. In general, TAM is recognized as an emerging field, experiencing substantial growth in publication volume over recent years. This increase may be attributed to significant advancements in immunology related to cancer therapy, which have garnered heightened interest in TAM research.

In terms of literature distribution by country, China was identified as the most productive contributor, with a total of 2169 articles on TAM, followed by the United States. The United States led in average citations, with 34.75 citations per article, underscoring its prominent position in the TAM field and extensive involvement in global scientific collaboration. This finding suggests that China may benefit from prioritizing the quality and clarity of its publications.

The analysis revealed that the most productive institutions were predominantly located in China, with Fudan University emerging as the leading institution in terms of published articles. The institutional collaboration network indicated a tendency for institutions to partner with universities within their own countries, likely due to the convenience and frequency of domestic interactions among scientific institutions.

Interdisciplinary collaboration across countries and institutions has played a pivotal role in advancing research in this domain. The CiteSpace overlay map highlights strong interdisciplinary connections, particularly between fields such as “molecular biology and immunology” and “medicine, healthcare, and clinical practice.” Basic research in these areas provides critical foundational support for translational and applied research efforts. The integration of emerging interdisciplinary research trends, particularly in conjunction with technological advancements such as single-cell RNA sequencing and gene editing, has accelerated progress in TAM research by enabling more precise characterization of TAM functions across various tumor types. These advancements contribute to the development of personalized therapeutic strategies. Strengthening interdisciplinary and international collaboration remains essential for achieving breakthroughs in tumor immunotherapy and enhancing the clinical translation of research findings.

Furthermore, an analysis of authors highlighted that the top 20 authors were all from China, indicating the significant role of Chinese researchers in this field. The timeline of author publications revealed a gradual increase in both publications and citations, suggesting that TAM has the potential to evolve into a major research focus.

Cancer immunotherapy has revolutionized the therapeutic landscape and has shown efficacy across various tumor types in numerous clinical cases. However, while some individuals benefit from this treatment, the majority do not respond, underscoring the need for new strategies to stimulate the immune system against cancer. Current immunotherapy approaches primarily focus on enhancing the functional properties of endogenous cytotoxic CD8 + T cells or injecting engineered T cells into patients. T cells are not the only immune components that affect tumor progression (positive or negative). Other immune components, including macrophages, also represent important therapeutic targets deserving further exploration [41].

Macrophages are typically the most abundant immune cells within tumors, and recent studies have demonstrated that specific macrophage subpopulations may influence tumor progression and response to treatment [18, 42, 43]. An analysis of keywords related to TAM research has identified key areas of focus and potential future directions. Frequently occurring keywords such as “expression,” “macrophage,” “cancer,” “cell,” “progression,” “activation,” “inflammation,” and “microenvironment” not only reflect the academic community’s recognition of TAM’s as critical components of tumor biology but also guide emerging clinical applications and translational research.

The keywords “expression” and “macrophage” emphasize the heterogeneity and functional diversity of TAMs within the TME. Advances in single-cell omics technologies enable high-resolution mapping of gene expression profiles across macrophage subtypes, facilitating detailed molecular characterization and the identification of therapeutic targets. This approach contributes to the development of personalized immunotherapies, including strategies that selectively inhibit pro-tumor TAM subsets while enhancing the activity of anti-tumor macrophage populations in specific tumor types [44].

The keywords “progression” and “activation” further highlight the dual role of macrophages in tumor development, as certain TAM subtypes promote tumor growth and metastasis, whereas others enhance anti-tumor immune responses [8]. A deeper understanding of these mechanisms is essential for the development of novel therapeutic strategies, such as combining immune checkpoint inhibitors with TAM-targeting agents to achieve synergistic effects.

Additionally, the keywords “inflammation” and “microenvironment underscore the influence of inflammatory responses within the TME on TAM function. Given that chronic inflammation plays a significant role in tumor development [45], interventions aimed at modulating the inflammatory state within the TME—whether through anti-inflammatory therapies or lifestyle modifications—may indirectly regulate TAM activity and phenotype, thereby improving clinical outcomes. This interdisciplinary approach exemplifies the principles of translational medicine by facilitating the transition from fundamental scientific discoveries to clinical applications.

The analysis of these keywords highlights the translational potential of TAM research in oncology, necessitating both a deeper understanding of TAM biology and the clinical validation of TAM-targeting therapeutic strategies to enhance cancer treatment options. Future research may prioritize the identification of TAM-associated biomarkers for early cancer detection and the development of precision therapies tailored to specific TAM subtypes.

Furthermore, this study examined highly-quoted articles across different time periods, demonstrating the contributions of scholars from diverse fields to the evolution of TAM research. With advancements in technologies, such as single-cell omics, researchers have gained a deeper understanding of macrophage biology, setting the stage for breakthroughs in current therapeutic approaches [46].

The understanding of TAMs has evolved significantly over the past 25 years [47]. TAMs were first identified about 50 years ago as immune cells capable of killing and phagocytosing cancer cells, and acting as anti-tumor agents [48]. However, subsequent studies revealed that TAMs can also promote tumor growth. It has been discovered that TME has the potential to convert the TAMs, or at least some of them, into cancer-proliferating cells, highlighting the need for further research into the biological complexity of tumor growth and metastasis [49]. Across a variety of cancers, an increased presence of TAMs has been associated with lower survival rates [50].

Despite the diverse functions of TAMs, their plasticity and heterogeneity present opportunities for therapeutic intervention. The identification of markers that distinguish tumor-promoting from anti-tumor TAMs has therefore become a critical area of research. Historically, TAM identification has relied on flow cytometry and histological methods, which utilize a limited set of markers and may insufficiently capture TAM diversity. The advent of single-cell omics technologies, particularly single-cell RNA sequencing (scRNA-seq), has significantly advanced the characterization of TAM heterogeneity by enabling the identification of distinct macrophage subpopulations and their functional states. Compared to bulk transcriptome sequencing, scRNA-seq provides high-resolution transcriptomic data at the single-cell level, offering deeper insights into cellular function and diversity [44].

In recent years, scRNA-seq has been applied to the study of most cancer types, facilitated by improved accessibility and an expanding repository of bioinformatics-derived data [51]. Despite its contributions to the field, scRNA-seq remains limited in its ability to analyze the spatial organization of tissues. The integration of spatial transcriptomics with scRNA-seq addresses this limitation by allowing the visualization of cellular RNA transcript distribution while preserving tissue structural integrity [52]. Spatial molecular omics plays a crucial role in defining positional relationships and intercellular interactions within tissues, providing insights into how spatial cell distribution influences disease pathogenesis [53].

Advancements in single-cell omics technologies have greatly enhanced the understanding of TAM molecular diversity, functional plasticity, and interactions with other components of the TME. Notably, these technologies have facilitated the identification of TAM subpopulations that may serve as potential biomarkers for disease progression and clinical outcomes. The complex interactions among various cell populations within tumors contribute to the formation of a distinct TME that supports tumor growth, metastasis, and resistance to therapy. Additionally, non-tumor cells within this environment exhibit unique transcriptional programs that influence tumor-associated heterogeneity, determining whether their activity supports or suppresses tumor progression. scRNA-seq has emerged as a powerful tool for dissecting cellular transcriptomes, enabling the identification of cell types and functional states that may be critical for predicting patient prognosis [54]. By revealing TAM subpopulations with specific genetic signatures, scRNA-seq has provided novel insights into macrophage heterogeneity in human cancers, offering potential avenues for targeted therapeutic strategies.

The critical role of TAMs in the TME has become increasingly evident, with significant advancements in understanding their basic mechanisms and clinical applications emerging as a major research focus over the past decade. TAMs differentiate into M1-like (anti-tumor) and M2-like (pro-tumor) phenotypes in response to distinct cytokine stimuli, with the integrin signaling pathway playing a key role in regulating their polarization [55]. Additionally, non-coding RNAs mediate interactions between tumor cells and macrophages, influencing tumor cell proliferation, migration, and apoptosis while also modulating immune cell differentiation [56]. Tumor cells further regulate macrophage polarization through exosomal transfer, establishing feedback mechanisms that significantly impact tumor progression, angiogenesis, and drug resistance.

Therapeutic strategies targeting TAMs primarily focus on inhibiting macrophage recruitment, reprogramming M2-like TAMs, depleting TAM populations, and utilizing TAMs for drug delivery [57]. These approaches have been extensively investigated, with nanotechnology emerging as a promising avenue for TAM-targeted therapy. Studies in nanomedicine suggest that modulating TAMs through specific molecular targets can enhance anti-tumor effects; however, challenges remain in achieving selective and efficient drug delivery to TAMs [58]. Given macrophages’ high phagocytic capacity, researchers have explored drug encapsulation in nanoparticles or liposomes to enable sustained release. Additionally, genetic engineering of macrophages to produce therapeutic proteins has emerged as a novel strategy for enhancing anti-tumor immune responses.

Metabolic regulation plays a fundamental role in TAM function, particularly glucose metabolism, which remains incompletely understood [59]. CD40, a member of the tumor necrosis factor receptor family, enhances anti-tumor immunity by stimulating antigen-presenting cell activation through interactions with T cells. Anti-CD40 antibodies exert tumor-suppressive effects by engaging tumor-infiltrating macrophages and increasing levels of CCL2 and IFNγ, thereby enhancing tumor cell elimination [60–62]. Within the TME, tumor endothelial cells contribute to cancer-associated angiogenesis, while lactic acid promotes M2-like polarization via GPR132, a process regulated by the PPARγ transcription factor, which inhibits GPR132 expression [24, 63]. Additionally, the mTOR signaling pathway serves as a key regulator of macrophage polarization and function [64] Certain chemotherapeutic agents exhibit dual effects by inducing tumor cell death while simultaneously depleting TAMs, thereby enhancing overall anti-tumor efficacy [65].

In conclusion, research on TAMs has provided novel insights into the complexity of the TME while highlighting potential directions for future anti-cancer therapeutic strategies. A deeper understanding of TAM function and regulatory mechanisms, combined with advancements in nanotechnology and genetic engineering, is expected to facilitate the optimization of TAM-targeted therapies and improve treatment outcomes.

Despite the significant progress in TAM research over the past decade, there are several challenges and unanswered questions. For instance, while TAM status and their response to therapy are correlated in some cases, the relationship between these states remains unclear. Moreover, tumor-promoting TAMs do not always exhibit M2-like phenotypes, indicating that defining TAM states beyond the M1/M2 dichotomy is important. Furthermore, the functional significance of TAM states recognized by scRNA-seq is still largely unknown. Existing macrophage-targeting therapies may also face limitations in their long-term efficacy, as they may not fully activate the adaptive immune system, increasing the risk of therapeutic resistance.

This study represents the first comprehensive bibliometric analysis of TAM research, highlighting major research themes and hotspots. Similar to other studies, this study has certain limitations. First, neither Scopus nor PubMed indexes only science-related literature, while only journals with an impact factor (IF) are included in the WoS database. It does exclude studies published in journals without an IF; however, this focus on high-impact journals ensures the inclusion of high-quality studies. Additionally, it has been proven that using the WoS database for bibliometric analysis provides more accurate results compared to other peer-reviewed scientific literature databases (e.g., Scopus and PubMed) [66]. Secondly, the analysis only considered articles published after 2013 in the WoS database, leaving out earlier studies, and non-English language articles were not included, which may have influenced the results. Nonetheless, the study offers valuable insights into the current state of TAM research.

## Summary

This study presents a comprehensive bibliometric analysis of the literature on TAM, offering insights into the academic structure, its historical development, and emerging trends in TAM research between 2013 and 2022. The analysis highlights the continued growth of TAM-related studies and identifies key contributors to the field. A significant shift in research methodology is noted, moving from traditional flow cytometry and histological techniques toward advanced single-cell omics approaches, which allow for a more nuanced understanding of TAM diversity.

Current research priorities include biomarkers, immunotherapy, TAM phenotypes, the TME, macrophage-targeted agents, and the response to therapy. These topics are expected to remain central to TAM research in the coming years. This analysis provides a valuable resource for readers who may not have extensive expertise in the field, offering them an accessible overview of recent developments. Moreover, this study aids researchers in identifying relevant publications, potential collaborators, and directions for further research.

## Supplementary Information

**Additional file 1.**

## Data Availability

The datasets generated during and/or analyzed during the current study are available from the corresponding author on reasonable request.

## References

[1] Wynn TA, Chawla A, Pollard JW. Macrophage biology in development, homeostasis and disease. Nature. 2013;496(7446):445–55. 10.1038/nature12034. 23619691 10.1038/nature12034 PMC3725458

[2] Pollard JW. Trophic macrophages in development and disease. Nat Rev Immunol. 2009;9(4):259–70. 10.1038/nri2528. 19282852 10.1038/nri2528 PMC3648866

[3] Geissmann F, Manz MG, Jung S, Sieweke MH, Merad M, Ley K. Development of monocytes, macrophages, and dendritic cells. Science. 2010;327(5966):656–61. 10.1126/science.1178331. 20133564 10.1126/science.1178331 PMC2887389

[4] Yan W, Li T, Yin T, Hou Z, Qu K, Wang N, et al. M2 macrophage-derived exosomes promote the c-KIT phenotype of vascular smooth muscle cells during vascular tissue repair after intravascular stent implantation. Theranostics. 2020;10(23):10712–28. 10.7150/thno.46143. 32929376 10.7150/thno.46143 PMC7482821

[5] Zhou T, Zheng Y, Sun L, Badea SR, Jin Y, Liu Y, et al. Microvascular endothelial cells engulf myelin debris and promote macrophage recruitment and fibrosis after neural injury. Nat Neurosci. 2019;22(3):421–35. 10.1038/s41593-018-0324-9. 30664769 10.1038/s41593-018-0324-9 PMC6913093

[6] Gordon S, Plüddemann A, Martinez EF. Macrophage heterogeneity in tissues: phenotypic diversity and functions. Immunol Rev. 2014;262(1):36–55. 10.1111/imr.12223. 25319326 10.1111/imr.12223 PMC4231239

[7] Erwig LP, Henson PM. Clearance of apoptotic cells by phagocytes. Cell Death Differ. 2008;15(2):243–50. 10.1038/sj.cdd.4402184. 17571081 10.1038/sj.cdd.4402184

[8] DeNardo DG, Ruffell B. Macrophages as regulators of tumour immunity and immunotherapy. Nat Rev Immunol. 2019;19(6):369–82. 10.1038/s41577-019-0127-6. 30718830 10.1038/s41577-019-0127-6 PMC7339861

[9] Ohri CM, Shikotra A, Green RH, Waller DA, Bradding P. Macrophages within NSCLC tumour islets are predominantly of a cytotoxic M1 phenotype associated with extended survival. Eur Respir J. 2009;33(1):118–26. 10.1183/09031936.00065708. 19118225 10.1183/09031936.00065708

[10] Ino Y, Yamazaki-Itoh R, Shimada K, Iwasaki M, Kosuge T, Kanai Y, et al. Immune cell infiltration as an indicator of the immune microenvironment of pancreatic cancer. Br J Cancer. 2013;108(4):914–23. 10.1038/bjc.2013.32. 23385730 10.1038/bjc.2013.32 PMC3590668

[11] Forssell J, Oberg A, Henriksson ML, Stenling R, Jung A, Palmqvist R. High macrophage infiltration along the tumor front correlates with improved survival in colon cancer. Clin Cancer Res. 2007;13(5):1472–9. 17332291 10.1158/1078-0432.CCR-06-2073

[12] Zheng JH, Nguyen VH, Jiang S-N, Park S-H, Tan W, Hong SH, et al. Two-step enhanced cancer immunotherapy with engineered secreting heterologous flagellin. Sci Transl Med. 2017. 10.1126/scitranslmed.aak9537. 28179508 10.1126/scitranslmed.aak9537

[13] Singh RK, Berry K, Matsushima K, Yasumoto K, Fidler IJ. Synergism between human monocyte chemotactic and activating factor and bacterial products for activation of tumoricidal properties in murine macrophages. J Immunol. 1993;151(5):2786–93. 8360492

[14] Lewis CE, Pollard JW. Distinct role of macrophages in different tumor microenvironments. Can Res. 2006;66(2):605–12. 10.1158/0008-5472.CAN-05-4005 16423985

[15] Gentles AJ, Newman AM, Liu CL, Bratman SV, Feng W, Kim D, et al. The prognostic landscape of genes and infiltrating immune cells across human cancers. Nat Med. 2015;21(8):938–45. 10.1038/nm.3909. 26193342 10.1038/nm.3909 PMC4852857

[16] Mantovani A, Sozzani S, Locati M, Allavena P, Sica A. Macrophage polarization: tumor-associated macrophages as a paradigm for polarized M2 mononuclear phagocytes. Trends Immunol. 2002;23(11):549–55. 12401408 10.1016/s1471-4906(02)02302-5

[17] Movahedi K, Laoui D, Gysemans C, Baeten M, Stangé G, Van den Bossche J, et al. Different tumor microenvironments contain functionally distinct subsets of macrophages derived from Ly6C(high) monocytes. Can Res. 2010;70(14):5728–39. 10.1158/0008-5472.CAN-09-4672. 10.1158/0008-5472.CAN-09-4672 20570887

[18] Mantovani A, Marchesi F, Malesci A, Laghi L, Allavena P. Tumour-associated macrophages as treatment targets in oncology. Nat Rev Clin Oncol. 2017;14(7):399–416. 10.1038/nrclinonc.2016.217. 28117416 10.1038/nrclinonc.2016.217 PMC5480600

[19] Wang B, Li Q, Qin L, Zhao S, Wang J, Chen X. Transition of tumor-associated macrophages from MHC class II(hi) to MHC class II(low) mediates tumor progression in mice. BMC Immunol. 2011;12:43. 10.1186/1471-2172-12-43. 21813021 10.1186/1471-2172-12-43 PMC3162940

[20] Liu M, O’Connor RS, Trefely S, Graham K, Snyder NW, Beatty GL. Metabolic rewiring of macrophages by CpG potentiates clearance of cancer cells and overcomes tumor-expressed CD47-mediated ‘don’t-eat-me’ signal. Nat Immunol. 2019;20(3):265–75. 10.1038/s41590-018-0292-y. 30664738 10.1038/s41590-018-0292-y PMC6380920

[21] Baer C, Squadrito ML, Laoui D, Thompson D, Hansen SK, Kiialainen A, et al. Suppression of microRNA activity amplifies IFN-γ-induced macrophage activation and promotes anti-tumour immunity. Nat Cell Biol. 2016;18(7):790–802. 10.1038/ncb3371. 27295554 10.1038/ncb3371

[22] Zhou W, Ke SQ, Huang Z, Flavahan W, Fang X, Paul J, et al. Periostin secreted by glioblastoma stem cells recruits M2 tumour-associated macrophages and promotes malignant growth. Nat Cell Biol. 2015;17(2):170–82. 10.1038/ncb3090. 25580734 10.1038/ncb3090 PMC4312504

[23] Ubil E, Caskey L, Holtzhausen A, Hunter D, Story C, Earp HS. Tumor-secreted Pros1 inhibits macrophage M1 polarization to reduce antitumor immune response. J Clin Invest. 2018;128(6):2356–69. 10.1172/JCI97354. 29708510 10.1172/JCI97354 PMC5983338

[24] Colegio OR, Chu N-Q, Szabo AL, Chu T, Rhebergen AM, Jairam V, et al. Functional polarization of tumour-associated macrophages by tumour-derived lactic acid. Nature. 2014;513(7519):559–63. 10.1038/nature13490. 25043024 10.1038/nature13490 PMC4301845

[25] Chen C, Hu Z, Liu S, Tseng H. Emerging trends in regenerative medicine: a scientometric analysis in CiteSpace. Expert Opin Biol Ther. 2012;12(5):593–608. 10.1517/14712598.2012.674507. 22443895 10.1517/14712598.2012.674507

[26] Carve M, Allinson G, Nugegoda D, Shimeta J. Trends in environmental and toxicity research on organic ultraviolet filters: a scientometric review. Sci Total Environ. 2021;773: 145628. 10.1016/j.scitotenv.2021.145628. 33940738 10.1016/j.scitotenv.2021.145628

[27] Chen X, Liu Y. Visualization analysis of high-speed railway research based on CiteSpace. Transp Pol. 2020;85:1–17. 10.1016/j.tranpol.2019.10.004.

[28] Sun J-Y, Zhang D, Wu S, Xu M, Zhou X, Lu X-J, et al. Resistance to PD-1/PD-L1 blockade cancer immunotherapy: mechanisms, predictive factors, and future perspectives. Biomark Res. 2020;8:35. 10.1186/s40364-020-00212-5. 32864132 10.1186/s40364-020-00212-5 PMC7450549

[29] Hou J, Yang X, Chen C. Measuring researchers’ potential scholarly impact with structural variations: four types of researchers in information science (1979–2018). PLoS ONE. 2020;15(6): e0234347. 10.1371/journal.pone.0234347. 32569295 10.1371/journal.pone.0234347 PMC7307741

[30] Mulet-Forteza C, Genovart-Balaguer J, Mauleon-Mendez E, Merigó JM. A bibliometric research in the tourism, leisure and hospitality fields. J Bus Res. 2019;101:819–27. 10.1016/j.jbusres.2018.12.002.

[31] Fortuna G, Aria M, Piscitelli A, Mignogna MD, Klasser GD. Global research trends in complex oral sensitivity disorder: a systematic bibliometric analysis of the structures of knowledge. J Oral Pathol Med. 2020;49(6):565–79. 10.1111/jop.13077. 32557908 10.1111/jop.13077

[32] Li W, Zhao Y, Wang Q, Zhou J. Twenty years of entropy research: a bibliometric overview. Entropy (Basel). 2019. 10.3390/e21070694. 33267408 10.3390/e21070694 PMC7515197

[33] Aria M, Alterisio A, Scandurra A, Pinelli C, D’Aniello B. The scholar’s best friend: research trends in dog cognitive and behavioral studies. Anim Cogn. 2021;24(3):541–53. 10.1007/s10071-020-01448-2. 33219880 10.1007/s10071-020-01448-2 PMC8128826

[34] Rodell M, Famiglietti JS, Wiese DN, Reager JT, Beaudoing HK, Landerer FW, et al. Author correction: emerging trends in global freshwater availability. Nature. 2019;565(7739):E7. 10.1038/s41586-018-0831-6. 30604767 10.1038/s41586-018-0831-6

[35] Hjørland B. Domain analysis in information science. J Doc. 2002;58(4):422–62. 10.1108/00220410210431136.

[36] Yao L, Hui L, Yang Z, Chen X, Xiao A. Freshwater microplastics pollution: detecting and visualizing emerging trends based on Citespace II. Chemosphere. 2020;245: 125627. 10.1016/j.chemosphere.2019.125627. 31864046 10.1016/j.chemosphere.2019.125627

[37] Gao Y, Shi S, Ma W, Chen J, Cai Y, Ge L, et al. Bibliometric analysis of global research on PD-1 and PD-L1 in the field of cancer. Int Immunopharmacol. 2019;72:374–84. 10.1016/j.intimp.2019.03.045. 31030093 10.1016/j.intimp.2019.03.045

[38] Gao H, Ding X-H, Wu S. Exploring the domain of open innovation: bibliometric and content analyses. J Clean Prod. 2020. 10.1016/j.jclepro.2020.122580.

[39] Zou X, Vu HL, Huang H. Fifty years of accident analysis & prevention: a bibliometric and scientometric overview. Accid Anal Prev. 2020;144: 105568. 10.1016/j.aap.2020.105568. 32562929 10.1016/j.aap.2020.105568

[40] Chen C, Leydesdorff L. Patterns of connections and movements in dual-map overlays: a new method of publication portfolio analysis. J Am Soc Inf Sci. 2014;65(2):334–51. 10.1002/asi.22968.

[41] Binnewies M, Roberts EW, Kersten K, Chan V, Fearon DF, Merad M, et al. Understanding the tumor immune microenvironment (TIME) for effective therapy. Nat Med. 2018;24(5):541–50. 10.1038/s41591-018-0014-x. 29686425 10.1038/s41591-018-0014-x PMC5998822

[42] Engblom C, Pfirschke C, Pittet MJ. The role of myeloid cells in cancer therapies. Nat Rev Cancer. 2016;16(7):447–62. 10.1038/nrc.2016.54. 27339708 10.1038/nrc.2016.54

[43] Bejarano L, Jordāo MJC, Joyce JA. Therapeutic Targeting of the Tumor Microenvironment. Cancer Discov. 2021;11(4):933–59. 10.1158/2159-8290.CD-20-1808. 33811125 10.1158/2159-8290.CD-20-1808

[44] Li XM, Wang CY. From bulk, single-cell to spatial RNA sequencing. Int J Oral Sci. 2021. 10.1038/s41368-021-00146-0. 34782601 10.1038/s41368-021-00146-0 PMC8593179

[45] Ferreira MT, Miyake JA, Gomes RN, Feitoza F, Stevannato PB, da Cunha AS, et al. Cyclooxygenase inhibition alters proliferative, migratory, and invasive properties of human glioblastoma cells in vitro. Int J Mol Sci. 2021. 10.3390/ijms22094297. 33919029 10.3390/ijms22094297 PMC8122446

[46] Wang Y, Zhang K, Li T, Maruf A, Qin X, Luo L, et al. Macrophage membrane functionalized biomimetic nanoparticles for targeted anti-atherosclerosis applications. Theranostics. 2021;11(1):164–80. 10.7150/thno.47841. 33391468 10.7150/thno.47841 PMC7681077

[47] Yin T, Li Y, Ren Y, Fuad ARM, Hu F, Du R, et al. Phagocytosis of polymeric nanoparticles aided activation of macrophages to increase atherosclerotic plaques in ApoE(-/-) mice. J Nanobiotechnol. 2021;19(1):121. 10.1186/s12951-021-00863-y. 10.1186/s12951-021-00863-y PMC8082811 33910571

[48] Evans R, Alexander P. Cooperation of immune lymphoid cells with macrophages in tumour immunity. Nature. 1970;228(5272):620–2. 5529055 10.1038/228620a0

[49] Qian B-Z, Pollard JW. Macrophage diversity enhances tumor progression and metastasis. Cell. 2010;141(1):39–51. 10.1016/j.cell.2010.03.014. 20371344 10.1016/j.cell.2010.03.014 PMC4994190

[50] Zhang Q-W, Liu L, Gong C-Y, Shi H-S, Zeng Y-H, Wang X-Z, et al. Prognostic significance of tumor-associated macrophages in solid tumor: a meta-analysis of the literature. PLoS ONE. 2012;7(12): e50946. 10.1371/journal.pone.0050946. 23284651 10.1371/journal.pone.0050946 PMC3532403

[51] Guruprasad P, Lee YG, Kim KH, Ruella M. The current landscape of single-cell transcriptomics for cancer immunotherapy. J Exp Med. 2021. 10.1084/jem.20201574. 33601414 10.1084/jem.20201574 PMC7754680

[52] Longo SK, Guo MG, Ji AL, Khavari PA. Integrating single-cell and spatial transcriptomics to elucidate intercellular tissue dynamics. Nat Rev Genet. 2021;22(10):627–44. 10.1038/s41576-021-00370-8. 34145435 10.1038/s41576-021-00370-8 PMC9888017

[53] Zhang LL, Chen DS, Song DL, Liu XX, Zhang YN, Xu X, et al. Clinical and translational values of spatial transcriptomics. Signal Transduct Target Ther. 2022. 10.1038/s41392-022-00960-w. 35365599 10.1038/s41392-022-00960-w PMC8972902

[54] Choi YH, Kim JK. Dissecting cellular heterogeneity using single-Cell RNA sequencing. Mol Cells. 2019;42(3):189–99. 30764602 10.14348/molcells.2019.2446 PMC6449718

[55] Dalpati N, Rai SK, Sharma P, Sarangi PP. Integrins and integrin-driven secretory pathways as multi-dimensional regulators of tumor-associated macrophage recruitment and reprogramming in tumor microenvironment. Matrix Biol. 2025;135:55–69. 10.1016/j.matbio.2024.12.003. 39645091 10.1016/j.matbio.2024.12.003

[56] Patni H, Chaudhary R, Kumar A. Unleashing nanotechnology to redefine tumor-associated macrophage dynamics and non-coding RNA crosstalk in breast cancer. Nanoscale. 2024;16(39):18274–94. 10.1039/d4nr02795g. 39292162 10.1039/d4nr02795g

[57] Kuznetsova AB, Kolesova EP, Parodi A, Zamyatnin AA, Egorova VS. Reprogramming tumor-associated macrophage using nanocarriers: new perspectives to halt cancer progression. Pharmaceutics. 2024. 10.3390/pharmaceutics16050636. 38794298 10.3390/pharmaceutics16050636 PMC11124960

[58] Kang XJ, Huang YZ, Wang HY, Jadhav S, Yue ZL, Tiwari AK, et al. Tumor-associated macrophage targeting of nanomedicines in cancer therapy. Pharmaceutics. 2024. 10.3390/pharmaceutics16010061. 38258072 10.3390/pharmaceutics16010061 PMC10819517

[59] Zhang X, Ji LL, Li MO. Control of tumor-associated macrophage responses by nutrient acquisition and metabolism. Immunity. 2023;56(1):14–31. 10.1016/j.immuni.2022.12.003. 36630912 10.1016/j.immuni.2022.12.003 PMC9839308

[60] Djureinovic D, Wang MN, Kluger HM. Agonistic CD40 antibodies in cancer treatment. Cancers. 2021. 10.3390/cancers13061302. 33804039 10.3390/cancers13061302 PMC8000216

[61] Beatty GL, Chiorean EG, Fishman MP, Saboury B, Teitelbaum UR, Sun WJ, et al. CD40 agonists alter tumor stroma and show efficacy against pancreatic carcinoma in mice and humans. Science. 2011;331(6024):1612–6. 10.1126/science.1198443. 21436454 10.1126/science.1198443 PMC3406187

[62] Long KB, Gladney WL, Tooker GM, Graham K, Fraietta JA, Beatty GL. IFNγ and CCL2 cooperate to redirect tumor-infiltrating monocytes to degrade fibrosis and enhance chemotherapy efficacy in pancreatic carcinoma. Cancer Discov. 2016;6(4):400–13. 10.1158/2159-8290.CD-15-1032. 26896096 10.1158/2159-8290.CD-15-1032 PMC4843521

[63] Chen PW, Zuo H, Xiong H, Kolar MJ, Chu Q, Saghatelian A, et al. Gpr132 sensing of lactate mediates tumor-macrophage interplay to promote breast cancer metastasis. Proc Natl Acad Sci USA. 2017;114(3):580–5. 10.1073/pnas.1614035114. 28049847 10.1073/pnas.1614035114 PMC5255630

[64] Weichhart T, Hengstschläger M, Linke M. Regulation of innate immune cell function by mTOR. Nat Rev Immunol. 2015;15(10):599–614. 10.1038/nri3901. 26403194 10.1038/nri3901 PMC6095456

[65] Baghdadi M, Wada H, Nakanishi S, Abe H, Han N, Putra WE, et al. Chemotherapy-induced IL34 enhances immunosuppression by tumor-associated macrophages and mediates survival of chemoresistant lung cancer cells. Can Res. 2016;76(20):6030–42. 10.1158/0008-5472.CAN-16-1170. 10.1158/0008-5472.CAN-16-1170 27550451

[66] Demir E, Akmeşe ÖF, Erbay H, Taylan-Özkan A, Mumcuoğlu KY. Bibliometric analysis of publications on house dust mites during 1980–2018. Allergol Immunopathol (Madr). 2020;48(4):374–83. 10.1016/j.aller.2020.01.001. 32284264 10.1016/j.aller.2020.01.001

