# Supplementary figures and images for: Global research trends in tumor-associated macrophage studies: a bibliometric analysis
Source: Discov Oncol. 2025 May 9;16:711. doi: 10.1007/s12672-025-02473-8 (PMC12064514; doi:10.1007/s12672-025-02473-8)

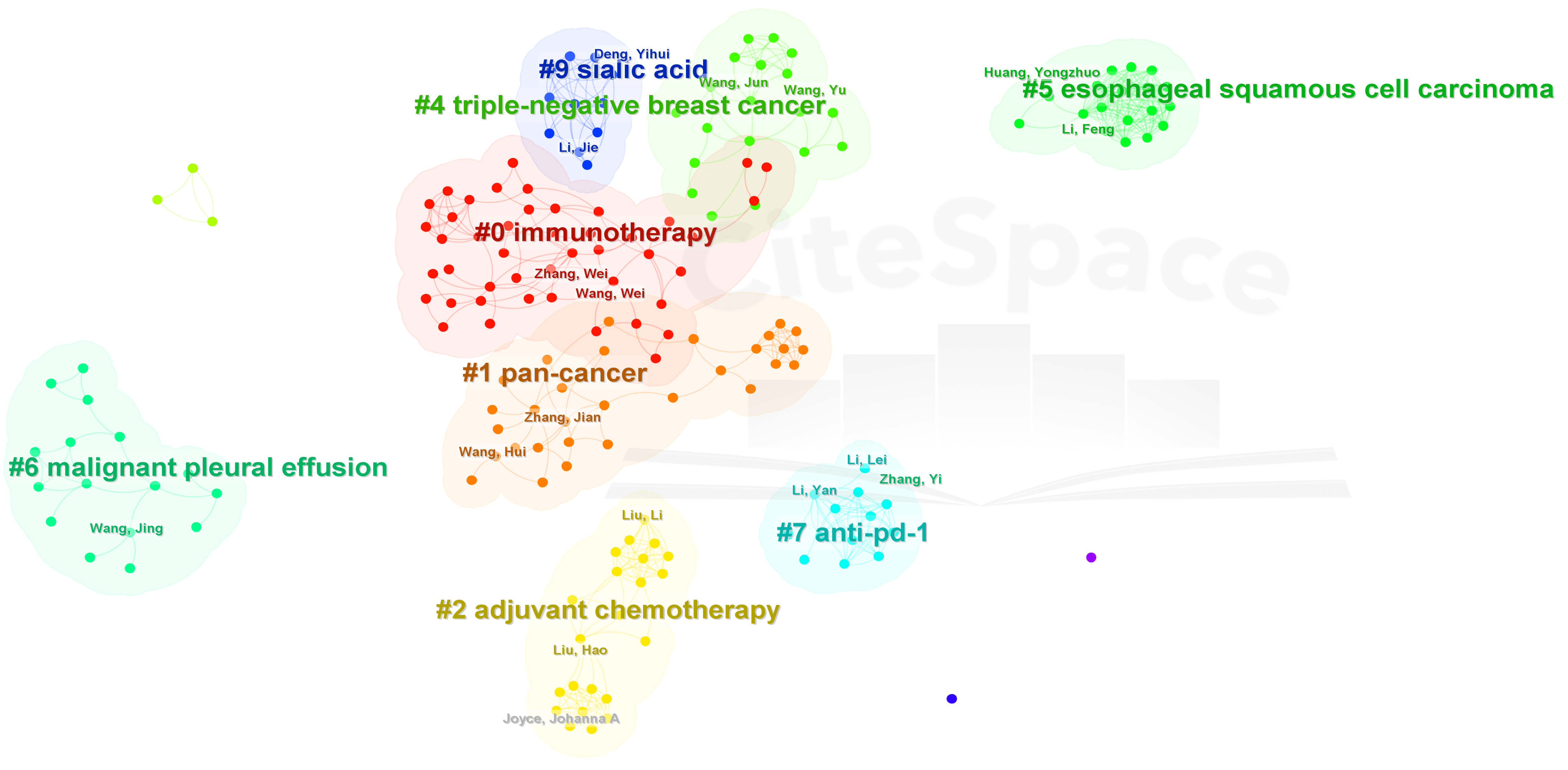

Supplement: Supplementary file 1 — Additional file 1. [file 12672_2025_2473_MOESM1_ESM.png]
